# Intrapulmonary migration of a fractured acupuncture needle: a case report

**DOI:** 10.1186/s13019-024-02502-7

**Published:** 2024-02-19

**Authors:** Lei Liu, Dong-Jie Ma, Ying-Zhi Qin, Hongsheng Liu

**Affiliations:** Department of Thoracic Surgery, Peking Union Medical College Hospital, Chinese Academy of Medical Sciences & Peking Union Medical College, Peking, 100730 P.R. China

**Keywords:** Case report, Acupuncture, Needle, Foreign body, Lung, Surgery

## Abstract

**Background:**

Acupuncture, a traditional Chinese medical treatment, has been gaining popularity over the years. However, it also presents certain risks. We report a case of a patient who discovered a foreign body in their lung several years after undergoing acupuncture.

**Case presentation:**

A middle-aged woman presented to our hospital with chest pain. An X-ray revealed a needle-like foreign body in the middle lobe of her right lung. The patient had previously undergone acupuncture treatment for local pain in her lower back and lower extremities many years prior. Based on the imaging findings and her medical history, we hypothesized that the foreign body in her lung was a result of a dislodged acupuncture needle. Through preoperative 3-dimensional reconstruction and indocyanine green localization, we were able to locate the foreign body in the lateral segment of the right middle lobe. We successfully removed the foreign body via wedge resection, and the patient made a smooth recovery post-surgery.

**Conclusion:**

Acupuncturists and surgeons should remain vigilant about the potential risks associated with acupuncture.

## Background

Acupuncture, a significant component of traditional Chinese medicine, involves the stimulation of specific skin points (acupuncture points) typically through the insertion of fine needles. While generally safe, acupuncture does carry some risks [1]. In this case report, we discuss a patient who experienced a needle dislodgement into the lung during acupuncture, necessitating surgical removal.

## Case presentation

**Fig. 1:**
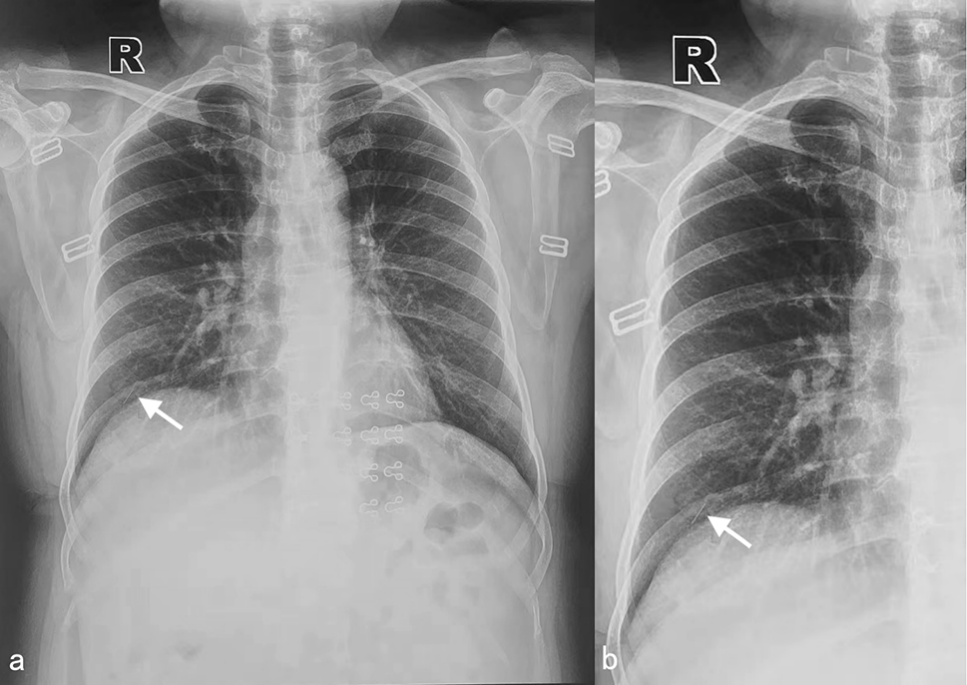
Preoperative X-ray examination: (**a**) isometric; (**b**) enlarged

**Fig. 2:**
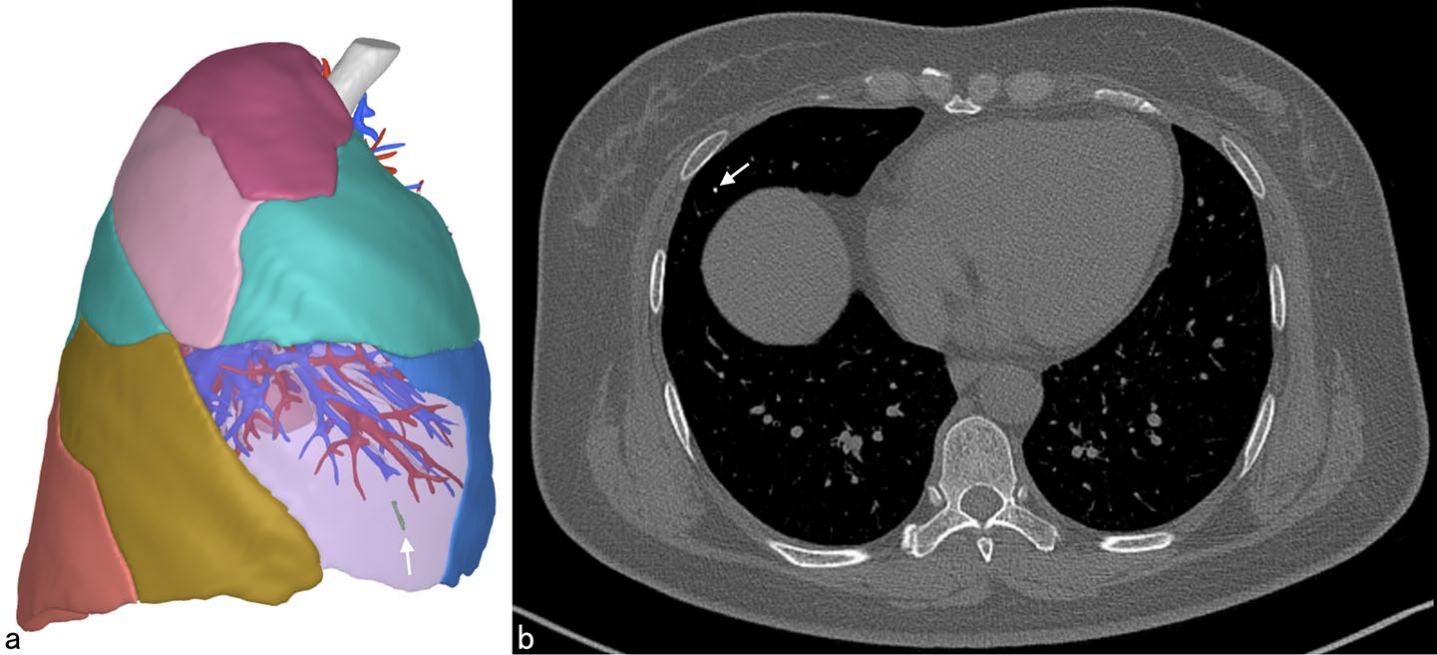
Preoperative imaging localization: (**a**) 3D reconstruction (white arrow); (**b**) CT scan (white arrow)

**Fig. 3:**
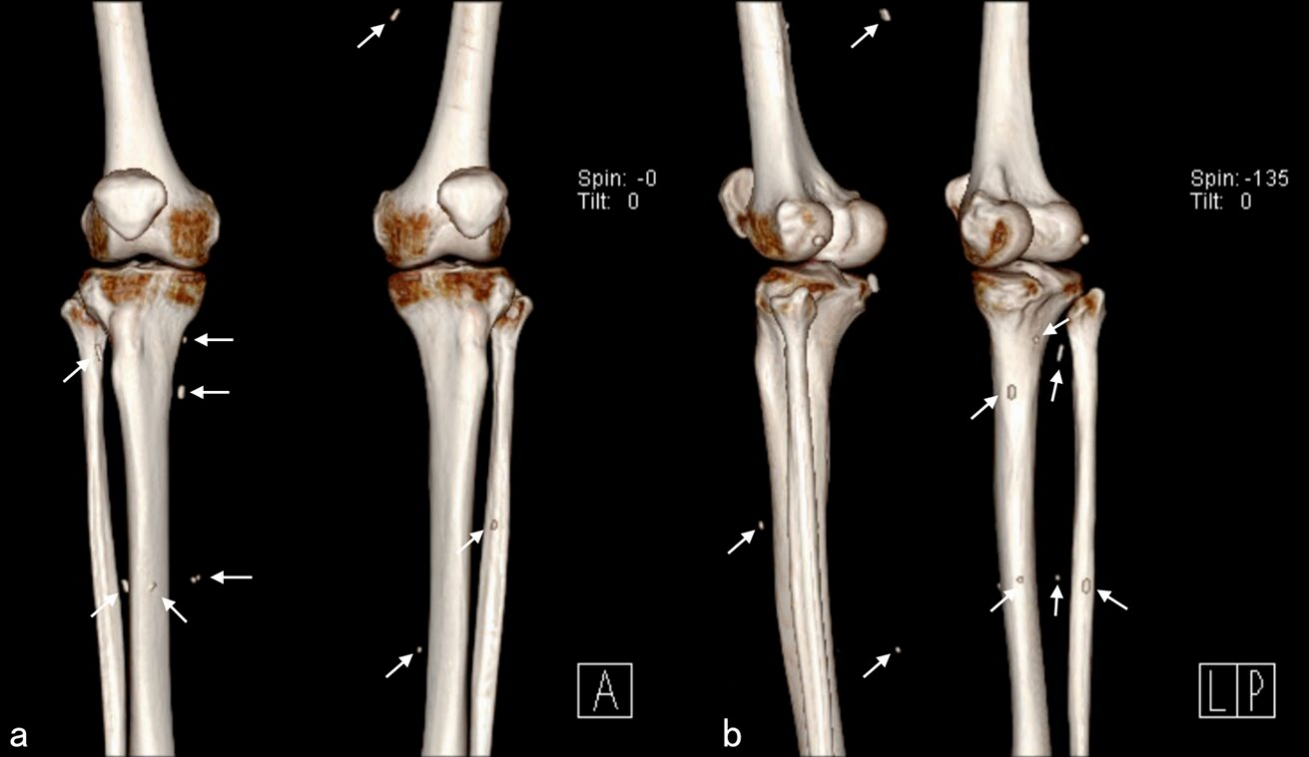
Multiple needle-shaped foreign bodies in the bilateral calves (white arrow)

A 49-year-old woman presented to our hospital with chest pain and a foreign body sensation in her right lung, persisting for two weeks. X-ray examination revealed a needle-like foreign body in the lateral area of the right lung (Fig. 1), and two similar foreign bodies near the right tibia and fibula. Subsequent chest computer tomography (CT) examination confirmed a needle-shaped foreign body, approximately 1 cm in length, in the lateral segment of the right middle lobe (Fig. 2). Multiple needle-shaped foreign bodies were also identified in the bilateral calves (Fig. 3). The patient had a history of unexplained syncope, but brain examination revealed no significant abnormalities. Other examinations and laboratory tests were also normal. Upon further medical history review, it was discovered that the patient had intermittently undergone acupuncture treatment for waist and leg pain since the age of 13. A needle-shaped foreign body was identified in the right calf over a decade ago, but only one was found and the related imaging data was lost. Given the patient’s current presentation and medical history, we hypothesized that the foreign body in the right lung’s middle lobe resulted from a broken acupuncture needle in the right calf that had entered the bloodstream.

**Fig. 4:**
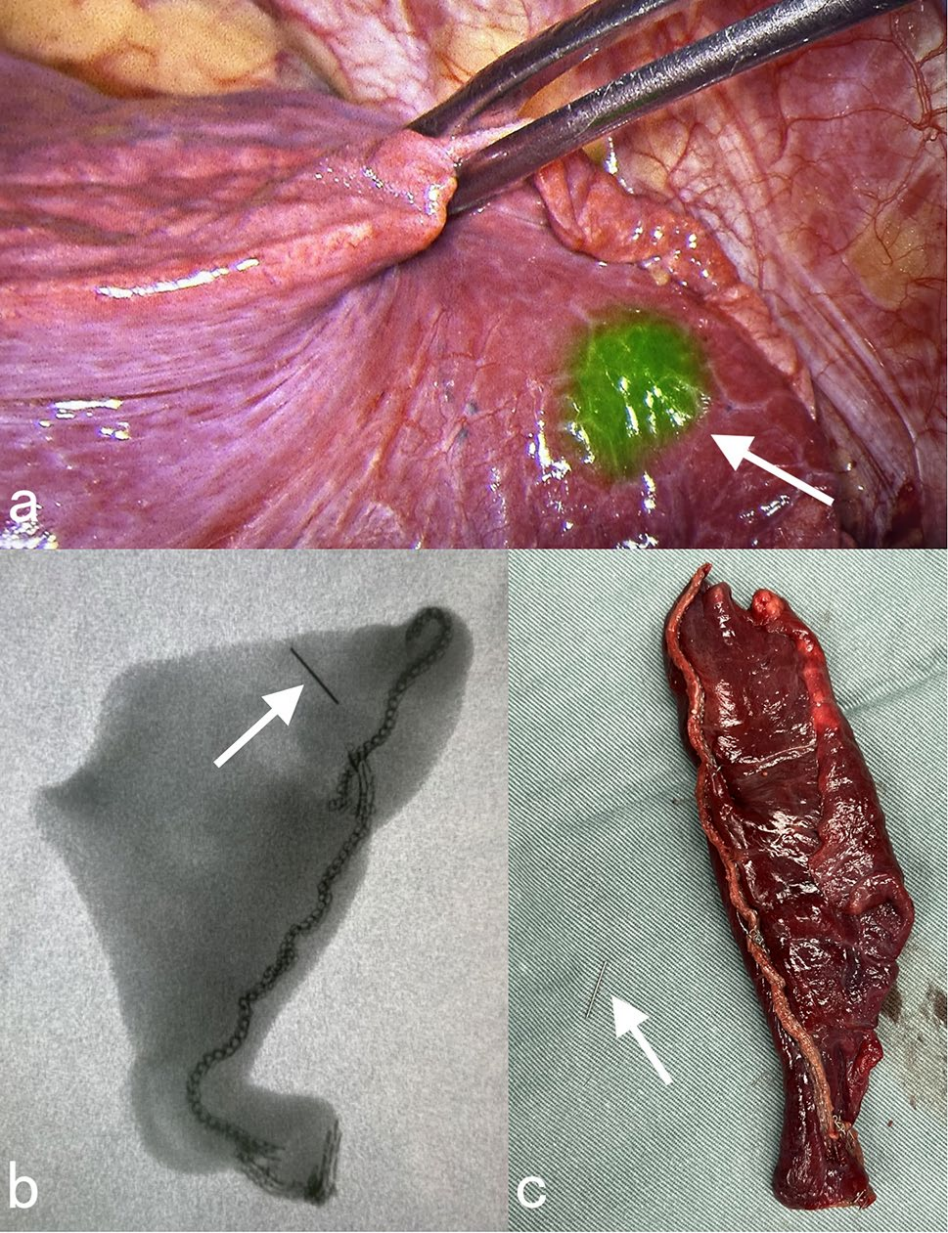
Intraoperative conditions: (**a**) indocyanine green localization area (white arrow); (**b**) X-ray perspective of resected lung tissue showed the location of the needle (white arrow); (**c**) removed needle (white arrow)

After thoroughly discussing the risks and benefits of surgery, the patient opted for the procedure. Preoperatively, we obtained orthopedic consultation, and the orthopedic specialist recommended initial conservative management given the relatively stable leg foreign body and the risks of iatrogenic nerve injury with attempted removal.Prior to surgery, we used indocyanine green for localization under CT guidance. During the operation, we performed a wedge resection of the right middle lobe using a video-assisted thoracic surgery (VATS) approach. The foreign body’s position was determined through intraoperative X-ray, leading to successful needle removal (Fig. 4). Intraoperatively, we utilized the indocyanine green localization spot as the center to calculate the resection margin based on the needle diameter. In total, approximately one-third of the right middle lobe was resected. We placed an intercostal drainage system, which was removed on postoperative day 2 due to minimal output. The patient’s postoperative recovery was uneventful. Postoperative pathology demonstrated chronic inflammatory changes in the lung parenchyma.

## Discussion

Acupuncture therapy, a gem of traditional Chinese medicine, is gaining global popularity and is currently employed to treat a variety of ailments including low back pain, neck pain, osteoarthritis/knee pain, and headaches [2]. Typically performed by professionally trained acupuncturists, the incidence of complications is relatively low, at approximately 8.6% [2]. Acupuncture involves the insertion of extremely thin needles, made from stainless steel, silver, or gold, into the subcutaneous soft tissues. Due to their delicate nature, these needles can easily break if mishandled. Furthermore, as acupuncture points are distributed throughout the body, complications can potentially occur in various regions.

Past reports have documented instances of foreign bodies in the lungs caused by acupuncture, but these were all due to direct migration from the acupuncture points [3–5]. In the case presented here, the patient did not receive acupuncture treatment on the chest or back, yet a needle was found in the lungs. Moreover, the acupuncture needles at the original site had fragmented from one long needle into several shorter ones. We hypothesize that the needle in the patient’s lungs originated from a broken acupuncture needle in the lower extremities, which entered the pulmonary circulation via the systemic circulation and lodged in the right middle lobe. We are astounded by the patient’s good fortune, as each step in this process could have been lethal. Given the critical nature of the lungs, we opted to surgically remove the needles.

Although the incidence of acupuncture-related complications is low according to the literature, it is not exceedingly rare. Wu et al. reported that injuries to internal organs, tissues, or nerves are the primary complications of acupuncture, with pneumothorax and central nervous system injuries being particularly notable [1]. Based on the case we have presented, we advocate for acupuncturists to exercise meticulous techniques during procedures and to check for needle breakage post-treatment. Concurrently, it is crucial to enhance patient education. Should a patient feel unwell following acupuncture treatment, they should seek immediate medical attention, and any broken needles within the body should be promptly removed to prevent serious complications.

## Conclusion

To the best of our knowledge, this is the first documented instance of an acupuncture needle breaking inside the body, traversing the systemic and pulmonary circulations, lodging in the lungs, and subsequently being successfully extracted via surgery. This case is exceedingly rare, and we anticipate that it will serve as a valuable reference for acupuncturists and surgeons in their future clinical practice.

## Data Availability

Data can be provided upon request.

## Abbreviations

CT: computer tomography
VATS: video-assistant thoracic surgery
